# Comparative evaluation of dynamic susceptibility contrast MRI techniques for brain imaging at 3T and 5T magnetic field strengths

**DOI:** 10.3389/fnhum.2026.1794651

**Published:** 2026-06-25

**Authors:** Pan Wang, Meng Gan, Liuyang Chen, Lixin Du, Zujun Hou, Cong Wang, Fan Lin

**Affiliations:** 1 Shantou University Medical College, Shantou, China; 2 Department of Radiology, Shenzhen Longhua District Central Hospital, Shenzhen, China; 3 School of Computer Engineering, Suzhou Polytechnic University, Suzhou, China; 4 FISCA Laboratory for Advanced Imaging, Nanjing, China; 5 School of Electronics and Information Engineering, Suzhou Polytechnic University, Suzhou, China; 6 Department of Radiology, The First Affiliated Hospital of Shenzhen University, Health Science Center, Shenzhen Second People's Hospital, Shenzhen, China

**Keywords:** cerebral blood flow, dynamic susceptibility contrast, functional imaging, high field MRI, microvascular perfusion

## Abstract

**Purpose:**

To evaluate the advantages of 5T magnetic resonance imaging (MRI) in dynamic susceptibility contrast (DSC) analysis for brain imaging and explore whether standardized perfusion parameters show similar distributions between 3T and 5T cohorts.

**Methods:**

Retrospective analysis of 52 adults undergoing 3T/5T MRI (July 2022–February 2025) with T1-weighted and DSC-MRI sequences. Perfusion parameters [cerebral blood volume (CBV), cerebral blood flow (CBF), and mean transit time (MTT)] and gray matter-to-white matter (GM/WM) ratios were quantified using MItalytics software. Signal-to-noise (SNR) and contrast ratios (CR) were compared, with Wilcoxon rank-sum tests.

**Results:**

Fifty-two patients (male/female: 24/28; age range: 24–74 years) were divided into 3T (*n* = 23) and 5T (*n* = 29) cohorts. In T1-weighted imaging, 3T demonstrated significantly higher SNR (*p* < 0.001), while 5T exhibited superior CR. For DSC-MRI, 5T achieved SNR comparable to 3T despite shorter repetition time (TR) and echo time (TE). No significant differences in perfusion parameters (CBV, CBF, MTT) were observed between field strengths. GM/WM ratios showed strong concordance: CBV ratios were 2.060 ± 0.223 (3T) vs. 2.062 ± 0.176 (5T), and CBF ratios were 1.985 ± 0.236 (3T) vs. 1.996 ± 0.173 (5T).

**Conclusion:**

In this retrospective analysis, 5T MRI showed higher T1-weighted contrast and similar DSC-MRI SNR relative to 3T under the tested protocols. DSC perfusion parameters, quantified using the MItalytics platform, showed no statistically significant between-cohort differences.

## 1 Introduction

Dynamic susceptibility contrast magnetic resonance imaging (DSC-MRI) is a pivotal tool for evaluating cerebral perfusion, enabling the quantification of hemodynamic parameters that correlate vascular pathophysiology with clinical outcomes (Śledzińska-Bebyn et al., 2025; Zhou et al., 2024; Mohammadi et al., 2025). By tracking the first-pass kinetics of intravascular contrast agents, DSC-MRI derives critical perfusion metrics–cerebral blood volume (CBV), cerebral blood flow (CBF), and mean transit time (MTT)–based on tracer kinetic theory (Kiselev, 2001). These parameters hold significant diagnostic and prognostic value across neurological disorders. In neuro-oncology, CBV mapping reliably distinguishes high-grade gliomas, which exhibit elevated microvascular proliferation and CBV values, from low-grade counterparts (Anzalone et al., 2018). Longitudinal DSC-MRI further aids in monitoring therapeutic response and predicting tumor recurrence (Patel et al., 2017; Quan et al., 2021). Similarly, in acute ischemic stroke, DSC-MRI underpins the perfusion-diffusion mismatch paradigm, delineating salvageable penumbral tissue from the infarct core to guide endovascular reperfusion therapy (Liu et al., 2021; Nael et al., 2019). Such applications underscore the necessity of robust perfusion quantification across clinical scenarios.

Higher magnetic field strengths offer several theoretical advantages for DSC-MRI (Shi et al., 2023; Harrison et al., 2024; Yin et al., 2024). The enhanced longitudinal magnetization at higher field strength enables improved signal-to-noise ratio (SNR) or higher spatial resolution, while the linear field-strength dependence of magnetic susceptibility effects amplifies contrast ratio (CR). This heightened susceptibility permits either reduced contrast agent (CA) doses or optimized imaging parameters, such as shorter repetition time (TR) or echo times (TE), without compromising contrast quality (Balchandani and Naidich, 2015; Burkett et al., 2021). Furthermore, the increased sensitivity to susceptibility-induced signal changes at ultrahigh fields may improve the detection of subtle perfusion alterations in pathophysiological states (Özütemiz et al., 2023).

However, these benefits are counterbalanced by technical challenges inherent to high-field systems. Elevated static field inhomogeneity and magnetic susceptibility gradients at air-tissue interfaces exacerbate off-resonance artifacts, manifesting as signal dropout and geometric distortions in echo-planar imaging (EPI) sequences. These artifacts predominantly affect the phase-encode direction, potentially compromising the accuracy of perfusion parameter quantification (Frayne et al., 2003; Testud et al., 2025). Additionally, radiofrequency field inhomogeneity and heightened specific absorption rate at higher field strength necessitate careful sequence optimization to maintain patient safety and image fidelity (Buntrock et al., 2025).

Despite growing clinical interest in 5T MRI, published evidence for brain imaging at 5T remains relatively limited compared with the larger 1.5T/3T literature (Yu et al., 2024; Lu et al., 2025). In addition, cross-field comparability of DSC-derived perfusion metrics (CBV/CBF/MTT) remains uncertain, because field-strength effects are often intertwined with vendor-specific acquisition settings and post-processing pipelines; this issue is also reflected by emerging 7T perfusion literature and cross-field analyses (Knutsson et al., 2017; Buntrock et al., 2025). Consequently, an explicit data-driven assessment is needed to clarify whether observed between-scanner differences are likely to reflect magnetic field effects, protocol effects, or both. Recent perfusion-focused studies and syntheses at conventional field strengths also emphasize that biomarker interpretation is sensitive to technique selection and analysis configuration (van Santwijk et al., 2022; Lavrova et al., 2022; Bayraktar et al., 2024), further motivating careful cross-field contextualization for 5T DSC-MRI. Additional recent high-field work, including 3T-vs.-7T comparative brain imaging and high-SNR 7T whole-brain quantification, further highlights active cross-field protocol development (Middlebrooks et al., 2024; Versteeg et al., 2024); meanwhile, emerging physics-informed DSC processing approaches indicate that estimated perfusion biomarkers remain sensitive to post-processing design choices (Rotkopf et al., 2024).

Against this background, the primary objective of this study was to perform a systematic comparison of DSC-MRI-derived perfusion parameters between 3T and 5T magnetic field strengths. Through quantitative analysis, we aimed to (1) evaluate potential 5T-related differences in DSC-MRI performance for brain imaging, including contrast dynamics, spatial resolution, and acquisition efficiency, and (2) assess whether standardized perfusion biomarkers (such as gray matter-to-white matter [GM/WM] ratios) showed similar values between cohorts.

## 2 Materials and methods

### 2.1 Study population

This study was approved by the institutional review board and patient informed consent was waived. A total of 52 patients underwent brain MR examination from July 2022 to February 2025 were enrolled. The inclusion criteria were: (1) adults aged 18 years or older; and (2) availability of a complete brain MRI protocol, including structural T1/T2-weighted imaging and DSC-MRI, acquired at either 3T or 5T. To ensure the accuracy of our automated SPM-based image processing and segmentation pipeline, specific exclusion criteria were applied. Patients were excluded if they had: (1) severe motion artifacts degrading image quality; (2) incomplete imaging data; or (3) massive space-occupying lesions, massive hemorrhages, or extensive territorial infarctions that caused severe midline shift or gross anatomical distortion, as these conditions predictably lead to the failure of automated spatial normalization and segmentation algorithms.

The baseline demographic and clinical characteristics of the 3T and 5T cohorts are summarized in Table 1. The 3T cohort comprised 23 patients (mean age 49.8 ± 14.8 years; 13 females), while the 5T cohort comprised 29 patients (mean age 42.3 ± 12.6 years; 18 females). There were no significant statistical differences between the two groups regarding age (*p* = 0.162) or sex distribution (*p* = 0.781). Furthermore, the overall distribution of pathological categories (Cerebrovascular/Ischemic, Tumors, Normal, and Other) was statistically comparable between the two cohorts (*p* = 0.514).

**Table 1:** Baseline demographic and clinical characteristics of the study cohorts.

Characteristic	Subgroup	3T (*n* = 23)	5T (*n* = 29)	*p*-value
Age (mean ± SD)		49.8 ± 14.8	42.3 ± 12.6	0.162
Age range		17–76	24–74	
Sex, *n* (%)				0.781
	Male	10 (43.5%)	11 (37.9%)	
	Female	13 (56.5%)	18 (62.1%)	
Diagnosis, *n*(%)				0.514
	Cerebrovascular/ischemic	10 (43.5%)	8 (27.6%)	
	Tumor/mass lesion	4 (17.4%)	7 (24.1%)	
	Normal/variant	2 (8.7%)	5 (17.2%)	
	Other (hemorrhage, calcification, etc.)	1 (4.3%)	5 (17.2%)	
	Missing	6 (26.1%)	4 (13.8%)	

### 2.2 MRI acquisition

MRI examinations were performed using 3T and 5T scanners equipped with phased-array head coils. Patients were positioned supine during imaging, and both structural T1-weighted sequences and DSC-MRI were acquired. Detailed imaging parameters are summarized in Table 2. For reproducibility, key DSC acquisition parameters are summarized here in addition to Table 2: TR/TE/flip angle were 1680 ms/30 ms/90° (Siemens 3T), 1593 ms/40 ms/75° (Philips 3T), and 1000 ms/15 ms/90° (5T); dynamic measurements were 60, 40, and 110, respectively. The corresponding temporal resolution per dynamic frame was therefore approximately 1.68 s, 1.59 s, and 1.00 s. For DSC-MRI, a gadolinium-based contrast agent (Gd-DOTA, 0.1 mmol/kg body weight) was administered intravenously via an MR-compatible power injector at 4 mL/s. No separate preload dose was administered before the DSC run. Synchronized with the contrast bolus injection, T2*-weighted gradient-echo echo-planar imaging (GRE-EPI) was performed to capture first-pass dynamics.

**Table 2:** Scanning parameters of MRI examination.

Sequence	Parameters	Device 1	Device 2	Device 3
T1	Equipment model	Siemens Skyra 3T	Philips Ingenia Elition X 3T	uMR Jupiter 5T
	Repetition time (ms)	2,000	2,000	1942
	Echo time (ms)	11	20	6.66
	Flip angle(degree)	150	120	90
	Thickness (mm)	4	4	5
	Field of view (mm^2^)	220 × 200	230 × 220	230 × 200
	Pixel spacing (mm)	0.69	0.45	0.31
DSC	Repetition time (ms)	1680	1593	1000
	Echo time (ms)	30	40	15
	Flip angle(degree)	90	75	90
	Thickness (mm)	4	4	5
	Field of view (cm^2^)	240 × 240	224 × 224	230 × 230
	Pixel spacing (mm)	1.88	1.75	1.80
	Measurements	60	40	110

### 2.3 Data analysis

#### 2.3.1 Brain tissue segmentation

The T1-weighted image was used for segmentation of the gray matter (GM), white matter (WM) and background regions. Thereafter, region masks were co-registered to the DSC images for the following analysis. The process was automatically implemented using the SPM (Penny et al., 2007).

To account for the differing native spatial resolutions and slice thicknesses between the 3T and 5T scanners, all perfusion maps and corresponding structural T1-weighted images were resampled to a common spatial resolution (1 × 1 × 5 mm^3^). To minimize partial volume effects (PVE), we applied a highly conservative probabilistic thresholding approach. Only voxels with a >95% probability of belonging to GM or WM were included in the final analytical masks. This strict thresholding effectively erodes the boundaries of the tissue masks, excluding tissue interfaces (e.g., GM/CSF or GM/WM boundaries) where PVE is most pronounced. Furthermore, rigorous quality control was integrated into the pipeline; an experienced radiologist visually inspected the coregistration and segmentation outputs for every subject to ensure accurate anatomical alignment and to manually exclude any cases with severe motion artifacts or registration failures. This largely automated pipeline is intended to improve processing consistency across subjects.

#### 2.3.2 DSC-MRI perfusion analysis

The DSC-MRI perfusion analyses were performed using the MItalytics postprocessing software (FISCA Healthcare). Details can be found as follows.

The change in transverse relaxation rate, which is proportional to the CA concentration, was calculated from the signal changes in the images induced by the CA bolus according to Equation 1.


$$\begin{array}{c} C\left(t\right)\propto \Delta R_{2}^{*}=-\left(\frac{1}{TE}\right)\ln\;\left(\frac{S\left(t\right)}{S\left(0\right)}\right), \end{array}$$ (1)

where *C*(*t*) is the concentration of CA at a certain timepoint *t*, $\Delta R_{2}^{*}$ is the transverse relaxation rate, TE is the echo time, *S*(*t*) is the signal at a certain timepoint *t* and *S*(0) is the averaged signal before the contrast arrival.

With calculated CA concentration, the arterial input function (AIF) was selected automatically by MItalytics using peak-height and time-to-peak criteria from candidate arterial voxels, and a single global AIF was then used per subject for perfusion quantification. To focus on first-pass hemodynamics and reduce recirculation effects, a gamma-variate function was fitted to the concentration-time curves (Boxerman et al., 1997). No additional model-based leakage-correction step (e.g., explicit post-bolus T1/T2^⋆^ leakage correction module) was applied in this pipeline; therefore, CBV/CBF values should be interpreted as relative DSC-derived metrics under this processing configuration.

The CBV and CBF were calculated using Equations 2, 3,


$$\begin{array}{c} \text{CBV}=\frac{\int_{0}^{\infty }C\left(t\right)dt}{\int_{0}^{\infty }C_{\text{AIF}\left(t\right)dt}}, \end{array}$$ (2)

$$\begin{array}{c} C_{t}\left(t\right)=\text{CBF}\cdot \left[R\left(t\right)*C_{\text{AIF}}\left(t\right)\right], \end{array}$$ (3)

where *C*_*t*_(*t*) is the concentration of CA in tissue at timepoint *t*, *C*_AIF_(*t*) is the concentration of CA in the AIF, *R*(*t*) is the tissue residue function, and * is the convolution operator. MTT is calclulated using Equation 4.


$$\begin{array}{c} \text{MTT}=\text{CBV}/\text{CBF}. \end{array}$$ (4)

#### 2.3.3 Qualitative image quality assessment

Two neuroradiologists (with 5 and 7 years of experience, respectively), who were blinded to the patients' clinical histories, independently evaluated the DSC-MRI images. Image quality was graded using a standard 5-point Likert scale: 1 = non-diagnostic (severe artifacts obscuring anatomy); 2 = suboptimal (moderate artifacts, limited diagnostic confidence); 3 = adequate (mild artifacts, sufficient diagnostic confidence); 4 = good (minimal artifacts, high diagnostic confidence); and 5 = excellent (no artifacts, outstanding diagnostic confidence). The average score of the two readers was used for final analysis. Inter-reader agreement was assessed using the intraclass correlation coefficient (ICC).

#### 2.3.4 Quantitative image analysis

To evaluate the quality of MR images from different devices, we use the SNR and CR as quantitative metrics. The SNR is defined as Equation 5,


$$\begin{array}{c} \text{SNR}=|\mu_{\text{region}}|/\sigma , \end{array}$$ (5)

where μ_region_ indicates the mean signal value of target regions, σ is the standard deviation of the background, regarded as noise. We emphasize that this background-noise-based SNR is an approximate, relative image-quality index (often termed “apparent SNR”) rather than an absolute physical SNR estimate. In modern parallel-imaging systems, spatially varying noise amplification (e.g., geometry-factor effects) can make background-noise estimation imperfect; therefore, SNR values in this study are interpreted primarily for within-study comparative purposes under the same analysis pipeline. CR is calculated as Equation 6,


$$\begin{array}{c} \text{CR}=\frac{\left|\mu_{\text{region1}}-\mu_{\text{region2}}\right|}{\left|\mu_{\text{region1}}+\mu_{\text{region2}}\right|}, \end{array}$$ (6)

where μ denotes the mean value.

### 2.4 Statistics

To assess the anatomical imaging quality, we quantitatively evaluated the SNR in GM and WM regions using T1-weighted imaging, along with calculating the CR between GM and WM tissues. These same quantitative metrics were subsequently applied to analyze the initial volume of the DSC-MRI acquisition. To prevent the heterogeneous mix of underlying pathologies (such as hypervascular tumors or ischemic penumbra) from confounding the perfusion analysis, quantitative measurements of CBF, CBV, and MTT were extracted from normal-appearing brain parenchyma.

For comparative analysis of DSC-MRI performance between 3T and 5T systems, we computed three key perfusion parameters: CBV, CBF, and MTT. Furthermore, we examined the GM/WM ratios for both CBV and CBF to identify potential between-cohort variations that may be related to field strength and/or protocol differences.

Statistical significance was determined using the Wilcoxon rank sum test, with a threshold of *p* < 0.05 indicating statistically significant differences. Due to the multiple hypothesis tests performed across different perfusion metrics and regions (Tables 3—5), nominal *p*-values between 0.04 and 0.05 were interpreted with caution. No formal family-wise error rate corrections (e.g., false discovery rate [FDR] or Bonferroni) were applied; therefore, findings reaching only borderline nominal significance are framed as exploratory. All computational analyses were performed using MATLAB (Version R2024a; The MathWorks, Inc., United States).

**Table 3:** Image quality metrics for anatomical structures at 3T and 5T.

Model	3T	5T	*p* value
T1 noise level	6.142 ± 1.418	18.823 ± 5.907	< 0.001
T1 SNR (GM)	57.005 ± 16.493	32.637 ± 7.095	< 0.001
T1 SNR (WM)	76.619 ± 19.209	46.728 ± 9.988	< 0.001
T1 CR (GM vs. WM)	0.144 ± 0.038	0.178 ± 0.015	< 0.001
DSC noise level	13.431 ± 4.408	31.968 ± 7.302	< 0.001
DSC SNR (GM)	66.785 ± 24.371	55.199 ± 12.037	0.097
DSC SNR (WM)	58.044 ± 22.870	50.425 ± 12.538	0.338
DSC CR (GM vs. WM)	0.0757 ± 0.0239	0.0510 ± 0.0177	< 0.001

**Table 4:** Perfusion parameters at 3T and 5T.

Model	3T	5T	*p* value
AIF peak	98.334 ± 46.879	163.682 ± 66.002	< 0.001
CBV	0.133 ± 0.051	0.112 ± 0.030	0.143
CBF	0.030 ± 0.014	0.023 ± 0.008	0.058
MTT	5.239 ± 2.537	5.293 ± 1.355	0.253
CBV(GM)	0.166 ± 0.058	0.140 ± 0.041	0.143
CBV(WM)	0.083 ± 0.037	0.068 ± 0.019	0.126
CBF(GM)	0.036 ± 0.017	0.028 ± 0.010	0.049
CBF(WM)	0.018 ± 0.008	0.014 ± 0.005	0.041
MTT(GM)	5.339 ± 2.635	5.328 ± 1.357	0.277
MTT(WM)	5.163 ± 2.518	5.154 ± 1.323	0.285
CBV(GM/WM)	2.060 ± 0.223	2.062 ± 0.176	0.927
CBF(GM/WM)	1.985 ± 0.236	1.996 ± 0.173	0.811

**Table 5:** Quantative metrics for different devices at 3T.

Device	Siemens (*n* = 18)	Philips (*n* = 5)	*p* value
T1 noise level	6.226 ± 1.478	5.838 ± 1.279	0.794
T1 SNR (GM)	62.801 ± 13.098	36.141 ± 8.462	0.002
T1 SNR (WM)	82.930 ± 15.455	53.900 ± 13.778	0.007
T1 CR (GM vs. WM)	0.130 ± 0.025	0.196 ± 0.029	0.002
DSC noise level	14.306 ± 4.605	10.281 ± 0.948	0.028
DSC SNR (GM)	57.190 ± 15.519	101.329 ± 18.360	0.002
DSC SNR (WM)	48.721 ± 14.343	91.603 ± 14.262	0.001
DSC CR (GM vs. WM)	0.083 ± 0.020	0.049 ± 0.018	0.007
AIF peak	110.372 ± 43.486	55.000 ± 32.416	0.028
CBV	0.125 ± 0.039	0.161 ± 0.080	0.352
CBF	0.032 ± 0.014	0.022 ± 0.009	0.233
MTT	4.621 ± 2.169	7.465 ± 2.735	0.023
CBV(GM)	0.158 ± 0.049	0.193 ± 0.086	0.434
CBV(WM)	0.075 ± 0.023	0.111 ± 0.063	0.314
CBF(GM)	0.039 ± 0.018	0.027 ± 0.010	0.192
CBF(WM)	0.019 ± 0.009	0.015 ± 0.006	0.456
MTT(GM)	4.692 ± 2.226	7.667 ± 2.909	0.028
MTT(WM)	4.524 ± 2.119	7.464 ± 2.709	0.023
CBV(GM/WM)	2.126 ± 0.180	1.825 ± 0.218	0.019
CBF(GM/WM)	2.041 ± 0.209	1.785 ± 0.239	0.019

## 3 Results

### 3.1 Data preprocessing and tissue segmentation

**Figure 1:**
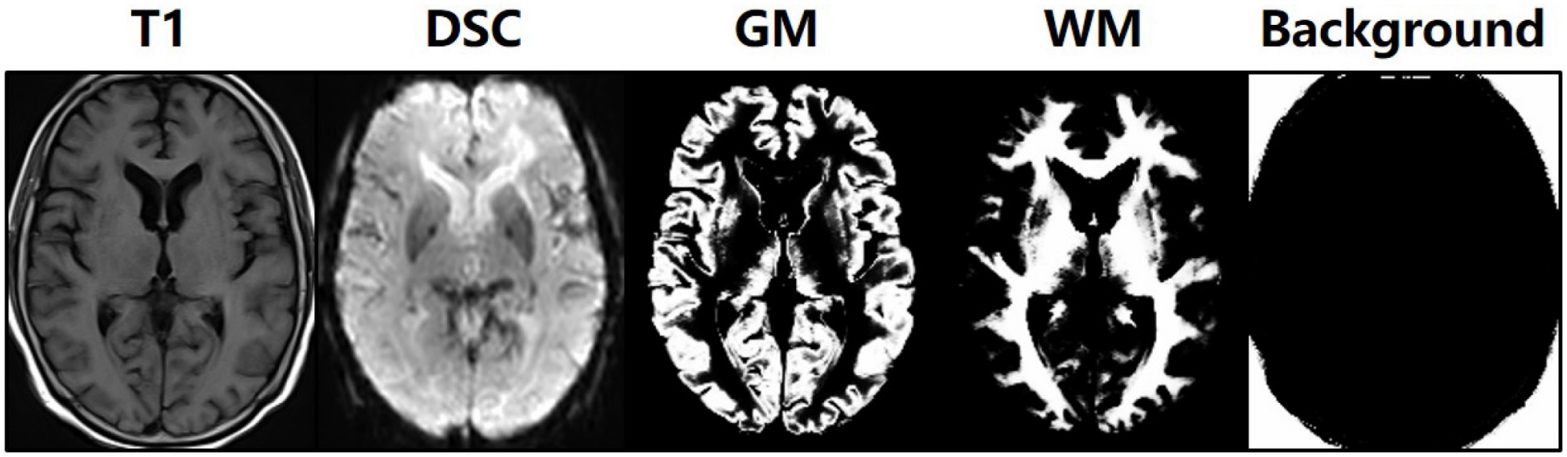
Representative imaging data and corresponding segmentation outcomes from a clinical participant.

Tissue segmentation was performed using T1-weighted imaging sequences owing to their superior spatial resolution and enhanced tissue contrast characteristics. To enable application of the derived segmentation masks to functional imaging data, each T1-weighted image was coregistered with DSC-MRI data prior to segmentation using a normalized mutual information algorithm. Representative segmentation outcomes are illustrated in Figure 1, demonstrating clear differentiation between GM, WM, and background regions in the segmented outputs.

Segmentation outputs were generated as probabilistic maps, with a conservative probability threshold of 0.95 applied to create binary tissue masks. This stringent thresholding approach ensured high confidence in voxel classification while maintaining anatomical continuity of the segmented structures. Quantitatively, all included examinations (52/52; 100%) completed the coregistration and segmentation workflow successfully, and no additional subjects were excluded during this post-processing stage after cohort enrollment (0/52).

### 3.2 Qualitative image quality scores

Inter-reader agreement for the qualitative image quality assessment was excellent, with an ICC of 0.84. Consistent with the quantitative findings showing comparable DSC-MRI SNR (Table 3), the subjective image quality scores demonstrated high diagnostic confidence for both systems. The mean image quality score was 4.2 ± 0.5 for the 5T cohort and 4.1 ± 0.6 for the 3T cohort, with no statistically significant difference (*p* = 0.42).

### 3.3 Comparative analysis of 3T Scanners

To evaluate the potential impact of hardware heterogeneity, we compared quantitative image quality and perfusion metrics between the Siemens and Philips platforms within the 3T cohort (Table 5). While Siemens scanners demonstrated higher T1-weighted SNR and Philips scanners showed higher DSC-weighted SNR, the primary physiological perfusion metrics, specifically CBV (*p* = 0.352) and CBF (*p* = 0.233), did not show statistically significant differences between the two vendors. Significant differences observed in MTT (*p* = 0.023) and AIF peak (*p* = 0.028) suggest variations in pulse-sequence optimization between the manufacturers. These findings indicate that image quality and temporal characteristics vary by vendor, whereas CBV and CBF did not differ significantly in this subgroup analysis.

### 3.4 Comparison of image quality at 3T and 5T in anatomical structures

**Figure 2:**
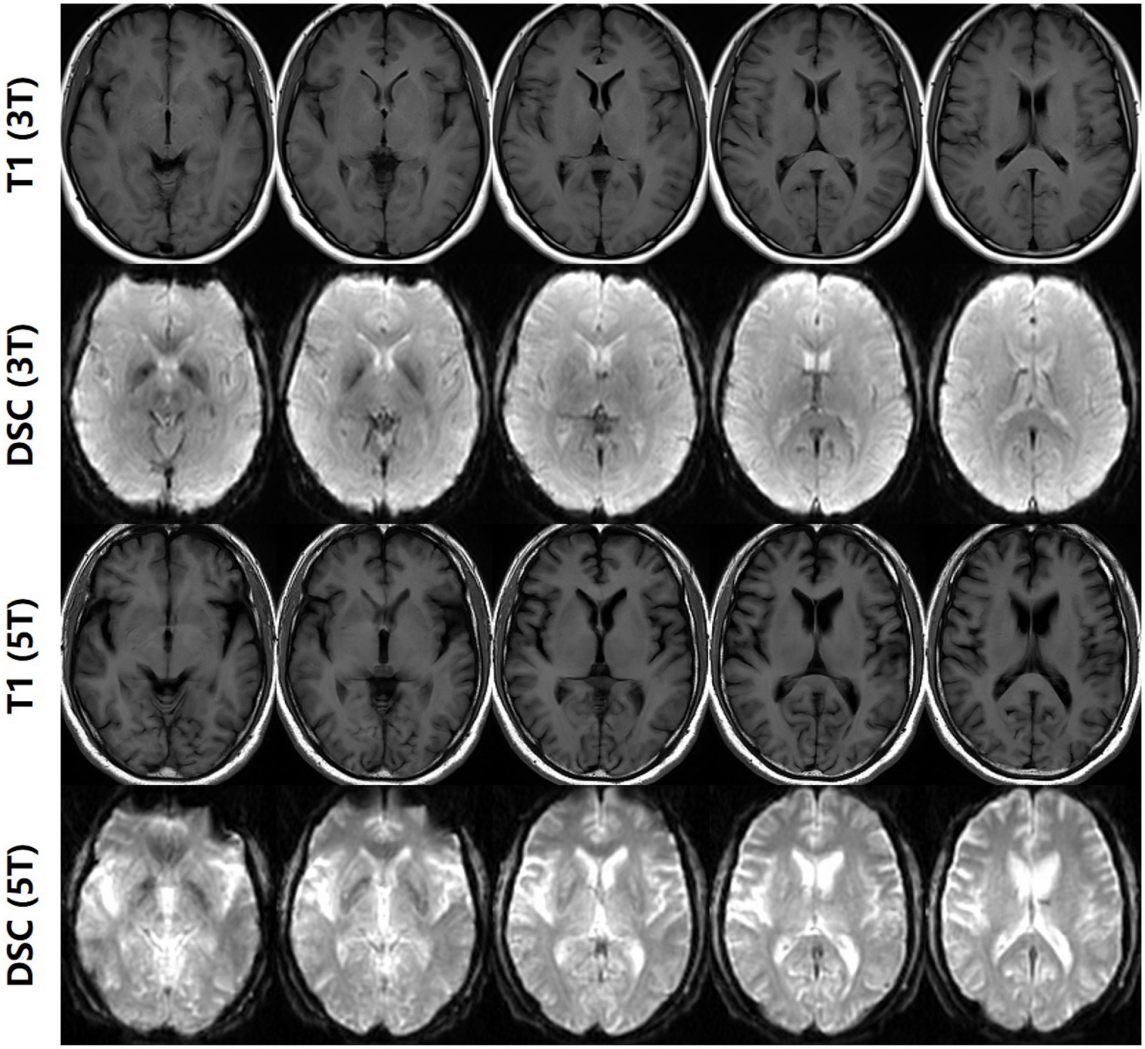
Demonstration of T1-weighted image and DSC-MRI image from 3T and 5T.

Figure 2 demonstrates representative T1-weighted anatomical images and DSC-MRI acquisitions from 3T and 5T systems. Qualitative assessment reveals enhanced boundary delineation between GM and WM structures in 5T T1-weighted images, particularly in cortical regions and subcortical nuclei. DSC-MRI sequences exhibited characteristic T2^⋆^-weighted GRE-EPI signal patterns, with cerebrospinal fluid-filled spaces such as ventricular systems showing high signal intensity at both field strengths, with increased conspicuity at 5T.

**Figure 3:**
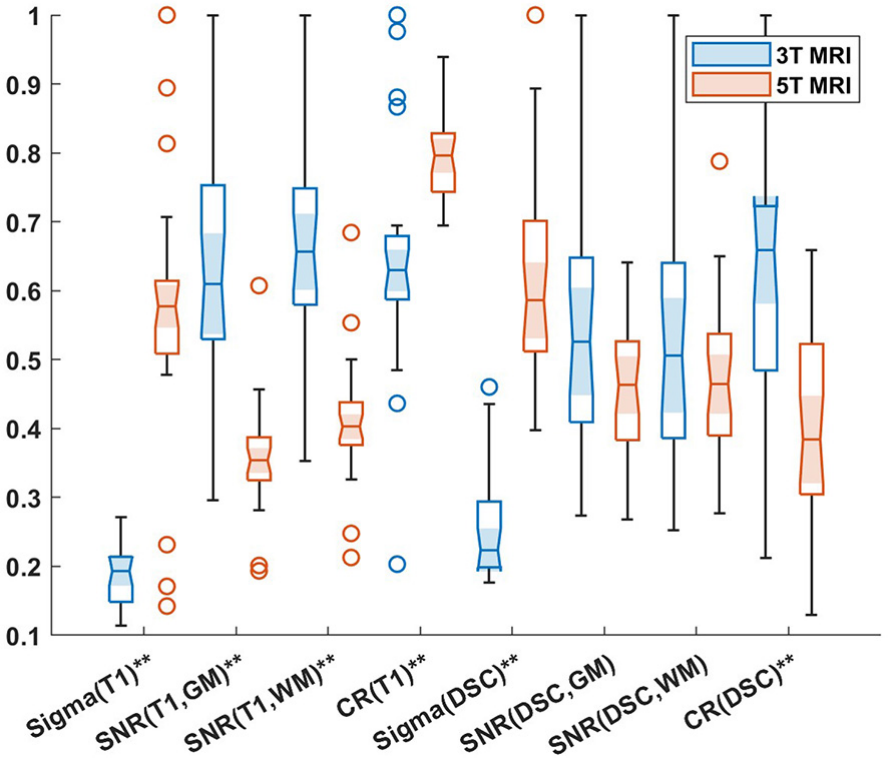
Boxplot of image quality metrics for anatomical structures (“**” indicates *p* < 0.01).

Quantitative analysis of five peri-central slices (Table 3) revealed significant field strength-dependent differences. 5T acquisitions demonstrated substantially elevated noise levels (18.823 ± 5.907) compared to 3T systems (6.142 ± 1.418, *p* < 0.001). Despite increased noise, 5T T1-weighted images exhibited superior GM/WM CR (0.178 ± 0.015 vs. 0.144 ± 0.038 at 3T, *p* < 0.001). DSC-MRI analysis showed comparable GM and SNR between field strengths, but significantly reduced contrast ratio in 5T acquisitions (0.0510 ± 0.0177) compared to 3T (0.0757 ± 0.0239, *p* < 0.001). These findings are visually corroborated by the distribution patterns in Figure 3.

### 3.5 Comparison of perfusion analysis results at 3T and 5T

**Figure 4:**
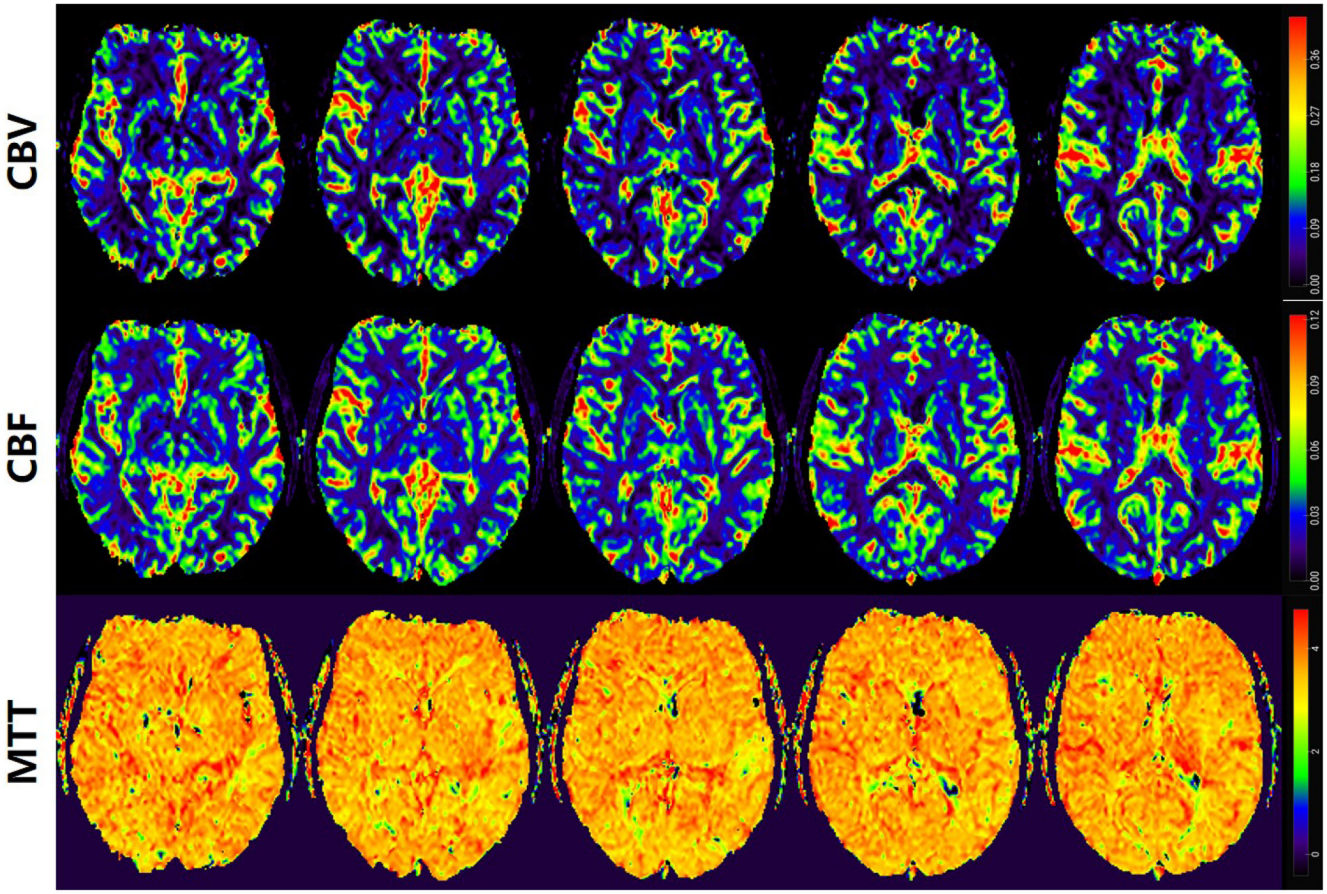
Demonstration of perfusion parameter maps at 3T.

**Figure 5:**
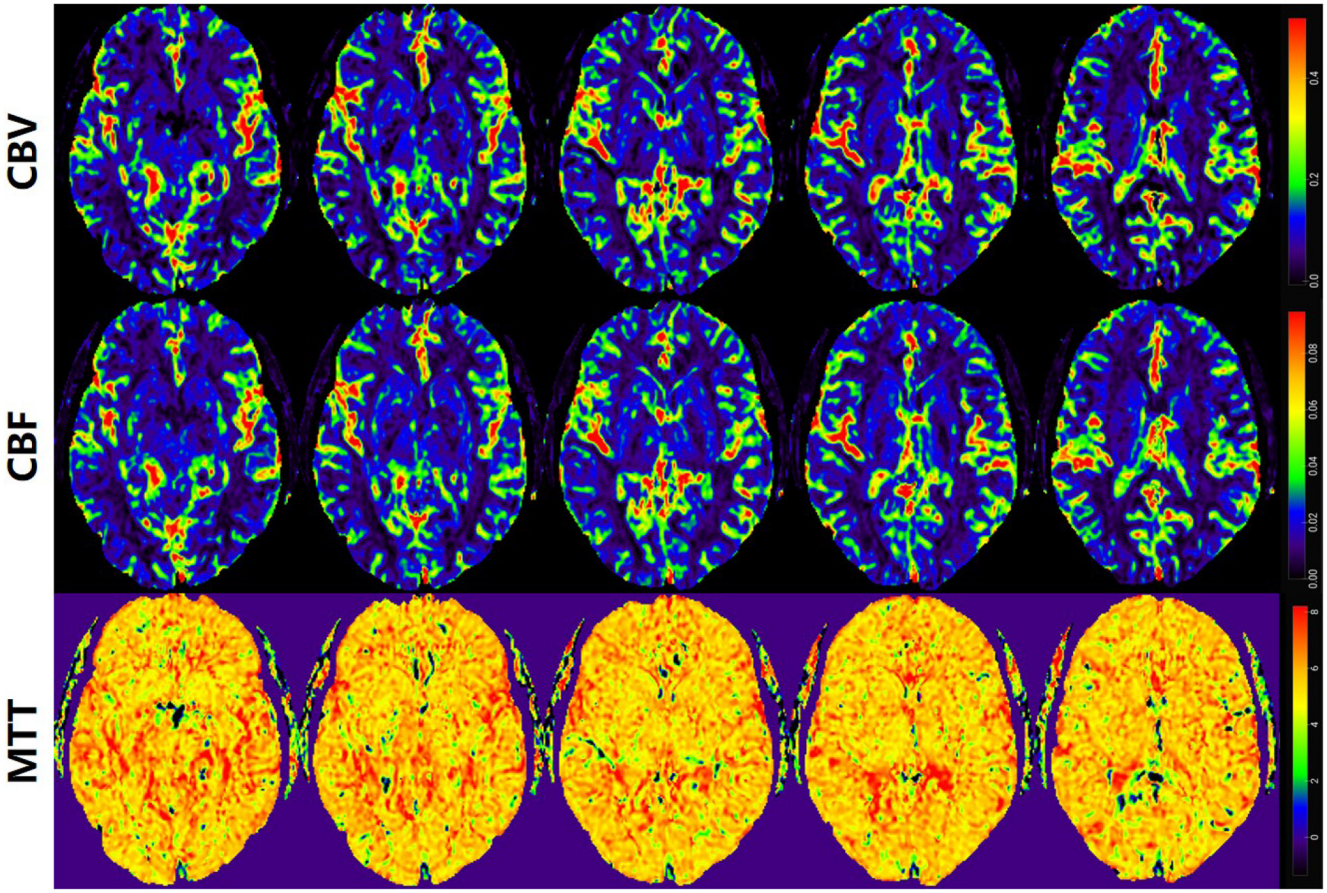
Demonstration of perfusion parameter maps at 5T.

Figures 4, 5 display representative DSC-MRI parameter maps acquired at 3T and 5T, respectively. While both field strengths demonstrate comparable image quality with preserved GM/WM contrast in CBV and CBF maps, the 5T acquisition notably maintains spatial fidelity without discernible susceptibility-related distortions.

**Figure 6:**
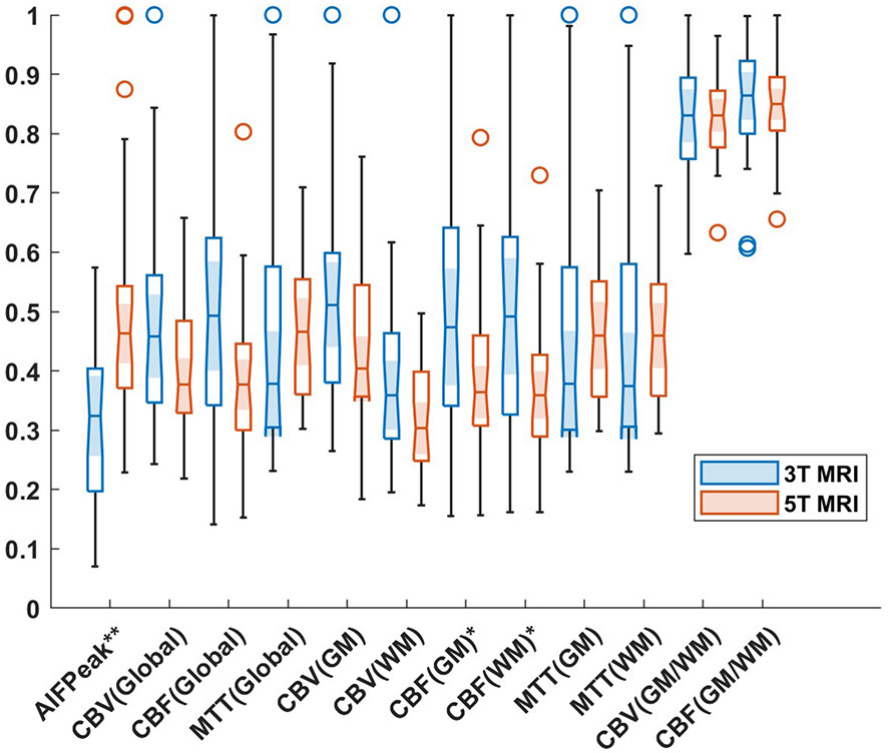
Boxplot of image quality metrics for perfusion parameters (“*” indicates *p* < 0.05, “**” indicates *p* < 0.01).

Table 4 summarizes quantitative comparisons (mean ± standard deviation) with corresponding distributions visualized in Figure 6. The AIF peak amplitude at 5T (163.682 ± 66.002) significantly exceeded the 3T value (98.334 ± 46.879, *p* < 0.001). While most perfusion parameters showed no statistically significant between-cohort differences, CBF in both gray matter (GM: *p* = 0.049) and white matter (WM: *p* = 0.041) approached borderline significance. Given the multiple comparisons performed, this finding is considered nominally significant and warrants further validation. MTT showed similar central tendencies between cohorts. Boxplot notch overlap analysis was used only as a descriptive visualization. Notably, GM/WM ratios for CBV and CBF remained similar between cohorts (*p* > 0.8), suggesting that these metrics may be less sensitive to cohort-level differences in this dataset. A mechanistic interpretation is that a larger 5T AIF peak does not necessarily translate into proportionally larger CBV/CBF values, because both CBV and deconvolution-based CBF are normalized to the AIF. In practice, sequence-dependent scaling factors (e.g., TE/TR/flip angle and temporal sampling differences) can increase the apparent amplitude of both arterial and tissue concentration curves. When tissue and AIF signals scale in parallel, the normalized perfusion estimates may remain relatively stable even when absolute AIF peak height differs.

The 5T data exhibited reduced variance across parameters (narrower standard deviations in Table 4 and compact boxplot distributions in Figure 6). This observation is descriptive; reduced dispersion alone should not be interpreted as definitive evidence of improved measurement precision or reliability without dedicated repeatability/reproducibility analysis.

## 4 Discussions

In this study, we conducted a comparative analysis of brain DSC-MRI at 3T and 5T field strengths. Our investigation focused on evaluating potential advantages of 5T MRI in DSC analysis for brain imaging using quantitative metrics, while also exploring whether standardized parameters show similar values between cohorts. Recent 5T brain MRI studies have demonstrated emerging clinical and network-level applications (Yu et al., 2024; Lu et al., 2025), but quantitative DSC cross-field comparability remains under-characterized, supporting the relevance of the present analysis.

In T1-weighted sequence, the 5T system demonstrated superior spatial resolution (0.31 mm) compared to 3T systems (0.69 mm and 0.45 mm). However, despite this advantage, 5T exhibited lower SNR, likely due to increased noise at higher field strengths. In terms of contrast performance, the 5T system achieved higher CR values under comparable TRs (~2,000 ms), highlighting its enhanced contrast generation capabilities. As for the DSC sequence, leveraging the higher field strength, 5T protocols utilized shorter TRs and TEs, enabling faster scan acquisition and elevated signal intensity. Notably, no significant SNR differences were observed between 3T and 5T in DSC sequences. The marginally lower CR at 5T in DSC-MRI may reflect trade-offs associated with reduced TR/TE settings.

The perfusion analysis demonstrated that 5T MRI systems achieved higher AIF peak compared to 3T systems, suggesting enhanced sensitivity for detecting subtle perfusion alterations. Comparative analysis revealed no statistically significant differences (α = 0.05) in most perfusion parameters. Notably, CBF measures approached but did not surpass conservative significance thresholds, with CBF(GM) = 0.049 and CBF(WM) = 0.041 (α = 0.05). Due to the multiple hypothesis tests performed across different tissue regions, isolated nominal *p*-values between 0.04 and 0.05 were interpreted with caution. We therefore treated these borderline findings as exploratory and limited interpretation to the observation that most tested perfusion parameters did not show statistically significant between-cohort differences. The GM/WM ratio demonstrated similar values across cohorts (*p*≈0.9), indicating potential as a candidate biomarker that may be relatively less sensitive to protocol variation; this interpretation requires confirmation in paired or harmonized studies. The apparently discrepant finding of higher AIF peak at 5T with largely unchanged CBV/CBF/MTT is physiologically and mathematically plausible in DSC processing. CBV is computed as a tissue-to-AIF integral ratio, and CBF is estimated by deconvolving tissue concentration with the AIF; both steps are therefore sensitive to relative scaling between tissue and arterial curves rather than AIF amplitude alone. In our non-harmonized cross-vendor setting, shorter TE/TR and different temporal resolution at 5T can increase bolus peak conspicuity, while parallel changes in tissue and AIF curves and first-pass modeling (gamma-variate fitting) can preserve normalized perfusion indices. This framework may reconcile the observed higher AIF peak with mostly non-significant between-cohort differences in downstream perfusion metrics.

5T-derived perfusion parameters exhibited reduced inter-subject variability compared to 3T in this dataset. This pattern may relate to a more conspicuous AIF during contrast bolus passage, although reduced variance alone should not be interpreted as definitive evidence of improved precision or reliability. Accurate AIF determination remains critical for minimizing errors in CBV and CBF calculations, as AIF inaccuracies propagate nonlinearly into parameter estimates (Østergaard et al., 1996).

Mean CBV values were 0.133 ± 0.051 (3T) and 0.112 ± 0.030 (5T), while CBF values measured 0.030 ± 0.014 (3T) and 0.023 ± 0.008 (5T). These semi-quantitative metrics reflect DSC-MRI's inherent limitations, including nonlinear signal-concentration relationships, AIF estimation uncertainties, and model-dependent assumptions (Van Osch et al., 2003; Starck et al., 2023). Absolute quantification requires complementary techniques like arterial spin labeling or PET (Naseri et al., 2024; Bhogal et al., 2025), yet DSC-MRI's relative parameters remain pivotal for assessing hemodynamic abnormalities in neuro-oncology and stroke (Abreu et al., 2024; Liu et al., 2025).

In this study, we observed that the GM/WM CBV and CBV ratios were 2.060 ± 0.223 and 1.985 ± 0.236 for 3T and 2.062 ± 0.176 and 1.996 ± 0.173 for 5T, which is in line with the literature. In Knutsson et al. (2017)'s study, the GM/WM CBV and CBF ratios were 2.0 and 2.4, respectively. In Bjørnerud and Emblem (2010)'s study, the ratios were 2.0 and 1.9, respectively. In a study by Wirestam et al. (2000), the GM/WM CBF ratio was 2.2 using standard singular value decomposition. The comprehensive PET study by Leenders et al. showed GM/WM ratios of 1.9 for CBV and 2.5 for CBF (Leenders et al., 1990). These external references support the physiological plausibility of our observed GM/WM ratios.

Since this study was a retrospective one utilizing clinical data, the MRI sequences were optimized by their respective vendors (Siemens/Philips for 3T; United Imaging for 5T) for routine diagnostic efficacy rather than cross-scanner harmonization. Consequently, there are substantial differences in sequence parameters, including TR/TE, spatial resolution, slice thickness, and the number of dynamic measurements. As T1 and T2^⋆^ relaxation times inherently change with higher magnetic fields, specific sequence adjustments are physically necessary; however, these differences directly affect baseline SNR, CR, and the dynamic temporal behavior of the AIF. Therefore, the observed differences reflect the synergistic effect of ultra-high-field physics combined with vendor-specific sequence optimizations.

Our study has several limitations. First, this was a retrospective non-paired cohort study, with different subjects scanned at 3T and 5T; therefore, between-group comparisons are susceptible to residual confounding and cannot substitute for within-subject field-strength comparisons. Second, the modest cohort size (*n* = 52) and limited exploration of DSC-MRI acquisition parameters restrict generalizability. Third, differing sequence timing parameters (TR/TE) between scanners complicate direct SNR and CR comparisons. Fourth, potential confounding effects from concurrent therapies (e.g., dexamethasone Quarles et al., 2005) may influence cerebral hemodynamics. Future investigations should employ: (1) longitudinal within-subject designs across field strengths, (2) multimodal validation with DCE-MRI/PET (Zheng et al., 2022; Long et al., 2025), and (3) histopathological correlation for treated lesion characterization (Boxerman et al., 2023). Such approaches would strengthen conclusions regarding 5T MRI's advantages in perfusion imaging.

In conclusion, this retrospective between-cohort comparison showed that 5T MRI achieved higher T1-weighted contrast resolution and similar DSC-MRI SNR relative to 3T despite shorter TR/TE. The heightened susceptibility effects at 5T were associated with more conspicuous AIF curves in this dataset, while most downstream perfusion metrics did not show statistically significant between-cohort differences. GM/WM ratios were similar between cohorts and may be useful candidate biomarkers for future harmonization studies. Overall, these findings support the potential of 5T MRI for high-resolution perfusion imaging and provide a foundation for future protocol optimization and clinical translation.

## Data Availability

The raw data supporting the conclusions of this article will be made available by the authors, without undue reservation.
